# Supplementary material for: Health-related quality of life of younger and older lower-income households in Malaysia
Source: PLoS One. 2022 Feb 8;17(2):e0263751. doi: 10.1371/journal.pone.0263751 (PMC8824345; doi:10.1371/journal.pone.0263751)
Supplement: S1 Table — (DOCX) [file pone.0263751.s001.docx]

**S1 Table. Characteristics of study sample**

| States | Municipalities | Estimated population size  (millions)^*^ | Number of study sample recruited |
| --- | --- | --- | --- |
| Selangor | Selayang  Ampang  Subang Jaya  Shah Alam  Petaling Jaya | 6.561 | 715 |
| Pahang | Kuantan | 1.683 | 190 |
| Sabah | Kota Kinabalu | 3.913 | 180 |
| Sarawak | Kuching Utara  Kuching Selatan  Padawan | 2.823 | 300 |
| Pulau Pinang | Seberang Perai | 1.777 | 380 |
| Johor | Johor Bahru | 3.795 | 360 |
| Total | | | 2125 |

^*^As of the first quarter of 2021
