# Supplementary material for: Health-related quality of life of younger and older lower-income households in Malaysia
Source: PLoS One. 2022 Feb 8;17(2):e0263751. doi: 10.1371/journal.pone.0263751 (PMC8824345; doi:10.1371/journal.pone.0263751)
Supplement: S2 Table — (DOCX) [file pone.0263751.s002.docx]

**S2 Table: Malay version of the EQ-5D-5L tool**

**EQ-5D-5L (Versi Melayu) / *EQ-5D-5L (Malay Version)***

Sila tandakan satu kotak yang menggambarkan keadaan kesihatan anda hari ini dengan paling tepat. / *Under each heading, please tick one box that best describes your health today.*

**PERGERAKAN / *MOBILITY***

Saya tidak menghadapi masalah untuk berjalan **[ ]**

*I have no problems in walking about* **[ ]**

Saya menghadapi sedikit masalah untuk berjalan **[ ]**

*I have slight problems in walking about* **[ ]**

Saya menghadapi masalah yang sederhana untuk berjalan **[ ]**

*I have moderate problems in walking about* **[ ]**

Saya menghadapi masalah yang teruk untuk berjalan **[ ]**

*I have severe problems in walking about* **[ ]**

Saya tidak berupaya untuk berjalan **[ ]**

*I am unable to walk about* **[ ]**

**PENJAGAAN DIRI / *SELF-CARE***

Saya tidak menghadapi masalah untuk membersihkan diri atau memakai

sendiri pakaian saya **[ ]**

*I have no problems washing or dressing myself* **[ ]**

Saya menghadapi sedikit masalah untuk membersihkan diri atau memakai

sendiri pakaian saya **[ ]**

*I have slight problems washing or dressing myself* **[ ]**

Saya menghadapi masalah yang sederhana untuk membersihkan diri atau

memakai sendiri pakaian saya **[ ]**

*I have some problems washing or dressing myself* **[ ]**

Saya menghadapi masalah yang teruk untuk membersihkan diri atau memakai

sendiri pakaian saya **[ ]**

*I have severe problems washing or dressing myself* **[ ]**

Saya tidak berupaya untuk membersihkan diri atau memakai sendiri pakaian saya **[ ]**

*I am unable to wash or dress myself* **[ ]**

**AKTIVITI-AKTIVITI BIASA (misalnya bekerja, belajar, membuat kerja rumah, aktiviti-aktiviti keluarga atau masa lapang) /**

***USUAL ACTIVITIES (e.g. work, study, housework, family or leisure activities)***

Saya tidak menghadapi masalah untuk melakukan aktiviti-aktiviti biasa saya **[ ]**

*I have no problems doing my usual activities* **[ ]**

Saya menghadap sedikit masalah untuk melakukan aktiviti-aktiviti biasa saya **[ ]**

*I have slight problems doing my usual activities* **[ ]**

Saya menghadapi masalah yang sederhana untuk melakukan aktiviti-aktiviti biasa saya **[ ]**

*I have some problems doing my usual activities* **[ ]**

Saya menghadapi masalah yang teruk untuk melakukan aktiviti-aktiviti biasa saya **[ ]**

*I have severe problems doing my usual activities* **[ ]**

Saya tidak berupaya untuk melakukan aktiviti-aktiviti biasa saya **[ ]**

*I am unable to do my usual activities* **[ ]**

**KESAKITAN / KETIDAKSELESAAN / *PAIN / DISCOMFORT***

Saya tidak berasa sakit atau tidak selesa **[ ]**

*I have no pain or discomfort* **[ ]**

Saya berasa sakit atau tidak selesa sedikit **[ ]**

*I have slight pain or discomfort* **[ ]**

Saya berasa sakit atau tidak selesa yang sederhana **[ ]**

*I have moderate pain or discomfort* **[ ]**

Saya berasa sakit atau tidak selesa yang teruk **[ ]**

*I have severe pain or discomfort* **[ ]**

Saya berasa sakit atau tidak selesa yang teramat sangat **[ ]**

*I have extreme pain or discomfort* **[ ]**

***ANXIETY / DEPRESSION***

Saya tidak berasa risau atau murung **[ ]**

*I am not anxious or depressed* **[ ]**

Saya berasa risau atau murung sedikit **[ ]**

*I am slightly anxious or depressed* **[ ]**

Saya berasa risau atau murung yang sederhana **[ ]**

*I am moderately anxious or depressed* **[ ]**

Saya berasa risau atau murung yang teruk **[ ]**

*I am severely anxious or depressed* **[ ]**

Saya berasa risau atau murung yang teramat sangat **[ ]**

*I am extremely anxious or depressed* **[ ]**

*
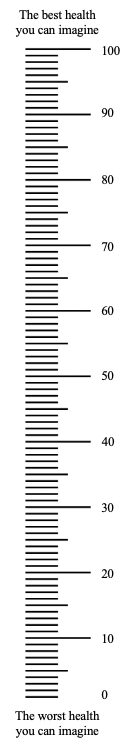
*

- Kami ingin tahu betapa baik atau tidak baik kesihatan anda pada HARI ini. */ We would like to know how good or bad your health is TODAY.*
- Skala ini bernombor dari 0 hingga 100. */ This scale is numbered from 0 to 100.*
- 100 bermaksud kesihatan paling baik yang boleh anda bayangkan. 0 bermaksud kesihatan paling teruk yang anda boleh bayangkan. */ 100 means the best health you can imagine. 0 means the worst health you can imagine.*
- Sila tandakan X pada skala untuk menandakan bagaimana kesihatan anda pada HARI INI. / *Mark an X on the scale to indicate how your health is TODAY.*
- Sekarang, sila tulis nombor yang anda tandakan pada skala di kotak di bawah. / *Now, please write the number you marked on the scale in the box below.*

KESIHATAN ANDA HARI INI =

*/ YOUR HEALTH TODAY =*
